# 2-Amino-6-methyl­pyridinium 4-hy­droxy­benzoate

**DOI:** 10.1107/S1600536813007939

**Published:** 2013-03-28

**Authors:** V. Kannan, P. Sugumar, S. Brahadeeswaran, M. N. Ponnuswamy

**Affiliations:** a Department of Physics, M.A.M. School of Engineering, Siruganur, Tiruchirappalli 621 105, India; b Centre of Advanced Study in Crystallography and Biophysics, University of Madras, Guindy Campus, Chennai 600 025, India; c Department of Physics, Anna University, BIT Campus, Tiruchirappalli 620 024, India

## Abstract

In the title mol­ecular salt, C_6_H_9_N_2_^+^·C_7_H_5_O_3_^−^, the dihedral angle between the benzene ring and the CO_2_ group in the anion is 6.1 (2)°. In the crystal, the cation and anion are linked by N—H⋯O and C—H⋯O hydrogen bonds, and the anions are connected by O—H⋯O hydrogen bonds, forming a three-dimensional network.

## Related literature

For general background to methyl­pyridinium derivatives, see: Blessing (1986 ▶); Brahadeeswaran *et al.* (2006 ▶); Brown (1976 ▶); Kvenvolden *et al.* (1971 ▶); Tomaru *et al.* (1991 ▶).
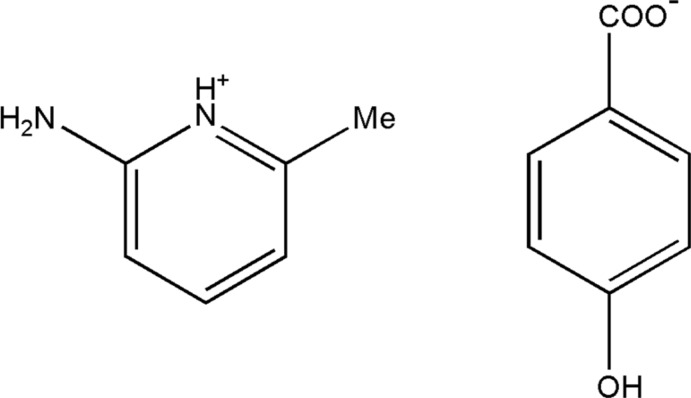


## Experimental

### 

#### Crystal data

C_6_H_9_N_2_^+^·C_7_H_5_O_3_^−^
*M* *_r_* = 246.26
Monoclinic, 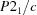

*a* = 11.9488 (3) Å
*b* = 9.2952 (3) Å
*c* = 12.4067 (3) Å
β = 117.116 (2)°
*V* = 1226.51 (6) Å^3^
*Z* = 4
Mo *K*α radiation
μ = 0.10 mm^−1^
*T* = 293 K
0.20 × 0.18 × 0.17 mm

#### Data collection

Bruker SMART APEXII CCD diffractometer
Absorption correction: multi-scan (*SADABS*; Bruker, 2008 ▶) *T*_min_ = 0.981, *T*_max_ = 0.984
11403 measured reflections
3084 independent reflections
2471 reflections with *I* > 2σ(*I*)
*R*_int_ = 0.023

#### Refinement

*R*[*F* ^2^ > 2σ(*F* ^2^)] = 0.041
*wR*(*F* ^2^) = 0.122
*S* = 1.04
3084 reflections
168 parameters
H atoms treated by a mixture of independent and constrained refinement
Δρ_max_ = 0.25 e Å^−3^
Δρ_min_ = −0.20 e Å^−3^

### 

Data collection: *APEX2* (Bruker, 2008 ▶); cell refinement: *SAINT* (Bruker, 2008 ▶); data reduction: *SAINT*; program(s) used to solve structure: *SHELXS97* (Sheldrick, 2008 ▶); program(s) used to refine structure: *SHELXL97* (Sheldrick, 2008 ▶); molecular graphics: *ORTEP-3* for Windows (Farrugia, 2012 ▶) and *DIAMOND* (Brandenburg, 1998 ▶); software used to prepare material for publication: *SHELXL97* and *PLATON* (Spek, 2009 ▶).

## Supplementary Material

Crystal structure: contains datablock(s) global, I. DOI: 10.1107/S1600536813007939/lx2273sup1.cif

Structure factors: contains datablock(s) I. DOI: 10.1107/S1600536813007939/lx2273Isup2.hkl

Supplementary material file. DOI: 10.1107/S1600536813007939/lx2273Isup3.cml

Additional supplementary materials:  crystallographic information; 3D view; checkCIF report

## Figures and Tables

**Table 1:** Hydrogen-bond geometry (Å, °)

*D*—H⋯*A*	*D*—H	H⋯*A*	*D*⋯*A*	*D*—H⋯*A*
N1—H1⋯O1^i^	0.86	2.00	2.8499 (13)	169
N2—H2*A*⋯O2^i^	0.86	1.94	2.7879 (14)	168
N2—H2*B*⋯O1^ii^	0.86	2.18	2.9902 (14)	157
O3—H3*A*⋯O2^iii^	0.97 (2)	1.67 (2)	2.6281 (14)	168.5 (19)
C4—H4⋯O3^iv^	0.93	2.51	3.4134 (17)	163

Symmetry codes: (i) 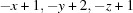; (ii) 
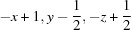
; (iii) 
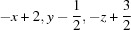
; (iv) 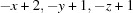.

**Fig. 1.**
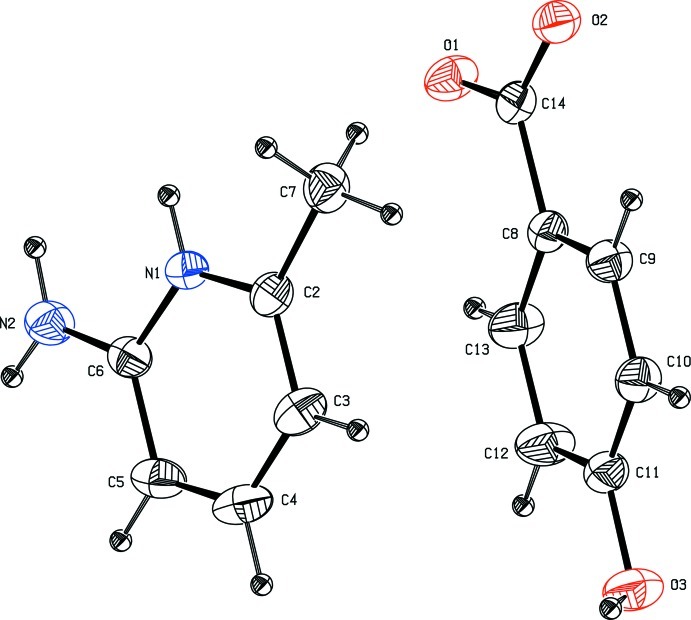
The molecular structure of the title compound with the atom numbering scheme. Displacement ellipsoids are drawn at the 30% probability level. H atoms are presented as small spheres of arbitrary radius.

**Fig. 2.**
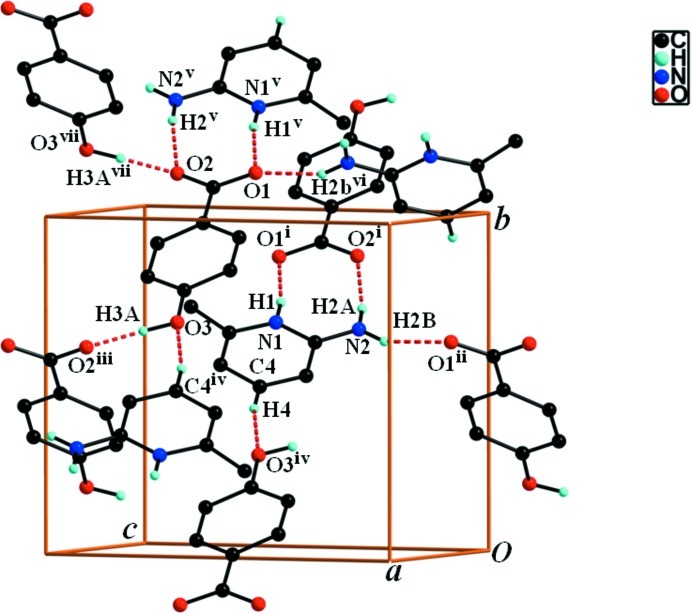
A view of the N—H···O, C—H···O and O—H···O hydrogen bonds (dotted lines) in the crystal structure of the title compound. H atoms not participating in hydrogen–bonding were omitted for clarity. [Symmetry code: (i) - x + 1, - y + 2, - z + 1; (ii) - x + 1 , y - 1/2, - z + 1/2; (iii) - x + 2, y - 1/2, - z + 3/2; (iv) - x + 2, - y + 1, - z + 1; (v) - x + 1, - y + 2, - z + 1; (vi) - x + 1, y + 1/2, - z + 1/2; (vii) - x + 2, y + 1/2, - z + 3/2.]

## supplementary crystallographic information

### Comment

Pyridine heterocycles and their derivatives are present in many large molecules having photo chemical, electro chemical and catalytic applications. Pyridine derivatives possess nonlinear optical (NLO) properties(Tomaru *et al.*, 1991). 4–*N*,*N*–dimethylamino–4'–*N*'–methyl stilbazolium tosylate (DAST) is used in generating and detecting terahertz (THz) frequencies (Brahadeeswaran *et al.*,2006). Carboxylic acids are believed to have existed in the prebiotic earth (Kvenvolden *et al.*, 1971) and form aggregation patterns. An attempt is made to solve the pyridine based crystal structures to explore the NLO behaviour.

The crystal structure of the title compound (Fig.1) consists of aminomethylpyridinium cation and hydroxybenzoate anion connected *via* N—H···O & C—H···O hydrogen bonds (Blessing,1986; Brown, 1976). The pyridinium ring is essentially planar, with a maximum deviation of -0.005 (1) Å for atom N1. The dihedral angle between the pyridinium ring in the cation and the benzene ring in the anion is 78.32 (7)°.

In the crystal structure (Fig. 2), the cation and anion are linked by N—H···O and C—H···O hydrogen bonds (Table 1), and the anions are connected by O—H···O hydrogen bonds (Table 1), forming a three–dimensional network.

### Experimental

Methanol solutions of 2–amino–6–methylpyridine (54 mg) and 4–hydroxybenzoic acid (69 mg) were mixed together and stirred for about 1 h to get a homogeneous mixture. The resulting solution was allowed to evaporate at 303 K slowly in a water bath which has a temperature accuracy of ± 0.01° C at ambient atmosphere. Brownish crystals with developed morphology of title compound were obtained after 15 days.

### Refinement

Especially about N—H & O—H according to RES. H atoms were positioned geometrically (N—H = 0.85–0.90 Å, O—H = 0.95–0.97 and C—H = 0.93–0.98Å) and allowed to ride on their parent atoms, with *U*_iso_(H) =1.5*U*_eq_(C) for methyl H 1.2*U*_eq_(C) for other H atoms.

### Crystal data

**Table:**

C_6_H_9_N_2_^+^·C_7_H_5_O_3_^−^	*F*(000) = 520
*M**_r_* = 246.26	*D*_x_ = 1.334 Mg m^−^^3^
Monoclinic, *P*2_1_/*c*	Mo *K*α radiation, λ = 0.71073 Å
Hall symbol: -P 2ybc	Cell parameters from 2671 reflections
*a* = 11.9488 (3) Å	θ = 1.9–28.4°
*b* = 9.2952 (3) Å	µ = 0.10 mm^−^^1^
*c* = 12.4067 (3) Å	*T* = 293 K
β = 117.116 (2)°	Block, white crystalline
*V* = 1226.51 (6) Å^3^	0.20 × 0.18 × 0.17 mm
*Z* = 4	

### Data collection

**Table:**

Bruker SMART APEXII CCD diffractometer	3084 independent reflections
Radiation source: fine-focus sealed tube	2471 reflections with *I* > 2σ(*I*)
Graphite monochromator	*R*_int_ = 0.023
ω and φ scans	θ_max_ = 28.4°, θ_min_ = 1.9°
Absorption correction: multi-scan (*SADABS*; Bruker, 2008)	*h* = −16→15
*T*_min_ = 0.981, *T*_max_ = 0.984	*k* = −10→12
11403 measured reflections	*l* = −16→15

### Refinement

**Table:**

Refinement on *F*^2^	Primary atom site location: structure-invariant direct methods
Least-squares matrix: full	Secondary atom site location: difference Fourier map
*R*[*F*^2^ > 2σ(*F*^2^)] = 0.041	Hydrogen site location: inferred from neighbouring sites
*wR*(*F*^2^) = 0.122	H atoms treated by a mixture of independent and constrained refinement
*S* = 1.04	*w* = 1/[σ^2^(*F*_o_^2^) + (0.0582*P*)^2^ + 0.2532*P*] where *P* = (*F*_o_^2^ + 2*F*_c_^2^)/3
3084 reflections	(Δ/σ)_max_ < 0.001
168 parameters	Δρ_max_ = 0.25 e Å^−^^3^
0 restraints	Δρ_min_ = −0.20 e Å^−^^3^

### Special details

**Table:**

Geometry. All esds (except the esd in the dihedral angle between two l.s. planes) are estimated using the full covariance matrix. The cell esds are taken into account individually in the estimation of esds in distances, angles and torsion angles; correlations between esds in cell parameters are only used when they are defined by crystal symmetry. An approximate (isotropic) treatment of cell esds is used for estimating esds involving l.s. planes.
Refinement. Refinement of F^2^ against ALL reflections. The weighted R-factor wR and goodness of fit S are based on F^2^, conventional R-factors R are based on F, with F set to zero for negative F^2^. The threshold expression of F^2^ > 2sigma(F^2^) is used only for calculating R-factors(gt) etc. and is not relevant to the choice of reflections for refinement. R-factors based on F^2^ are statistically about twice as large as those based on F, and R- factors based on ALL data will be even larger.

### Fractional atomic coordinates and isotropic or equivalent isotropic displacement parameters (Å^2^)

**Table:**

	*x*	*y*	*z*	*U*_iso_*/*U*_eq_	
C2	0.63520 (11)	0.65353 (14)	0.57200 (11)	0.0399 (3)	
C3	0.72343 (13)	0.56067 (17)	0.57177 (14)	0.0528 (3)	
H3	0.7947	0.5393	0.6434	0.063*	
C4	0.70557 (14)	0.49803 (16)	0.46291 (15)	0.0562 (4)	
H4	0.7652	0.4342	0.4623	0.067*	
C5	0.60208 (14)	0.52912 (14)	0.35780 (13)	0.0486 (3)	
H5	0.5912	0.4868	0.2857	0.058*	
C6	0.51139 (12)	0.62547 (13)	0.35806 (11)	0.0384 (3)	
C7	0.64299 (12)	0.72809 (17)	0.68095 (12)	0.0490 (3)	
H7A	0.5776	0.6934	0.6988	0.074*	
H7B	0.6331	0.8298	0.6660	0.074*	
H7C	0.7234	0.7094	0.7485	0.074*	
C8	0.81847 (10)	0.98339 (13)	0.59471 (10)	0.0370 (3)	
C9	0.90757 (11)	0.92379 (15)	0.70239 (11)	0.0415 (3)	
H9	0.9070	0.9489	0.7747	0.050*	
C10	0.99711 (12)	0.82801 (15)	0.70462 (11)	0.0433 (3)	
H10	1.0556	0.7891	0.7778	0.052*	
C11	0.99954 (11)	0.79001 (15)	0.59772 (11)	0.0435 (3)	
C12	0.91149 (14)	0.84879 (19)	0.48968 (12)	0.0592 (4)	
H12	0.9124	0.8242	0.4175	0.071*	
C13	0.82247 (13)	0.94363 (18)	0.48875 (12)	0.0542 (4)	
H13	0.7637	0.9819	0.4154	0.065*	
C14	0.72117 (10)	1.08629 (13)	0.59372 (10)	0.0369 (3)	
N1	0.53173 (9)	0.68267 (11)	0.46589 (8)	0.0359 (2)	
H1	0.4763	0.7405	0.4674	0.043*	
N2	0.40790 (11)	0.66254 (13)	0.26020 (9)	0.0482 (3)	
H2A	0.3555	0.7216	0.2661	0.058*	
H2B	0.3930	0.6276	0.1908	0.058*	
O1	0.63458 (8)	1.12882 (11)	0.49535 (8)	0.0503 (3)	
O2	0.73063 (8)	1.12516 (11)	0.69528 (8)	0.0474 (2)	
O3	1.08494 (10)	0.69729 (13)	0.59355 (10)	0.0598 (3)	
H3A	1.145 (2)	0.672 (2)	0.675 (2)	0.089 (6)*	

### Atomic displacement parameters (Å^2^)

**Table:**

	*U*^11^	*U*^22^	*U*^33^	*U*^12^	*U*^13^	*U*^23^
C2	0.0376 (6)	0.0431 (7)	0.0414 (6)	−0.0007 (5)	0.0200 (5)	0.0035 (5)
C3	0.0437 (7)	0.0584 (9)	0.0572 (8)	0.0125 (6)	0.0239 (6)	0.0110 (7)
C4	0.0607 (8)	0.0492 (8)	0.0751 (10)	0.0178 (7)	0.0453 (8)	0.0095 (7)
C5	0.0657 (8)	0.0392 (7)	0.0571 (8)	0.0039 (6)	0.0422 (7)	−0.0004 (6)
C6	0.0463 (6)	0.0351 (6)	0.0416 (6)	−0.0035 (5)	0.0269 (5)	−0.0008 (5)
C7	0.0444 (7)	0.0597 (8)	0.0395 (6)	−0.0010 (6)	0.0162 (5)	−0.0015 (6)
C8	0.0327 (5)	0.0395 (6)	0.0367 (6)	0.0008 (5)	0.0140 (5)	−0.0002 (5)
C9	0.0417 (6)	0.0474 (7)	0.0345 (6)	0.0047 (5)	0.0165 (5)	0.0002 (5)
C10	0.0390 (6)	0.0483 (7)	0.0358 (6)	0.0072 (5)	0.0110 (5)	0.0041 (5)
C11	0.0372 (6)	0.0472 (7)	0.0430 (6)	0.0062 (5)	0.0156 (5)	−0.0027 (5)
C12	0.0563 (8)	0.0815 (11)	0.0350 (6)	0.0240 (8)	0.0167 (6)	−0.0031 (7)
C13	0.0485 (7)	0.0720 (10)	0.0339 (6)	0.0205 (7)	0.0117 (5)	0.0031 (6)
C14	0.0328 (5)	0.0392 (6)	0.0391 (6)	−0.0015 (5)	0.0168 (5)	0.0015 (5)
N1	0.0373 (5)	0.0366 (5)	0.0379 (5)	0.0027 (4)	0.0208 (4)	0.0000 (4)
N2	0.0516 (6)	0.0566 (7)	0.0380 (5)	0.0035 (5)	0.0217 (5)	−0.0052 (5)
O1	0.0431 (5)	0.0630 (6)	0.0413 (5)	0.0166 (4)	0.0162 (4)	0.0076 (4)
O2	0.0415 (5)	0.0576 (6)	0.0425 (5)	0.0060 (4)	0.0188 (4)	−0.0047 (4)
O3	0.0539 (6)	0.0729 (7)	0.0498 (6)	0.0261 (5)	0.0212 (5)	0.0006 (5)

### Geometric parameters (Å, º)

**Table:**

C2—N1	1.3606 (15)	C8—C14	1.5013 (16)
C2—C3	1.3634 (18)	C9—C10	1.3825 (17)
C2—C7	1.4837 (18)	C9—H9	0.9300
C3—C4	1.395 (2)	C10—C11	1.3855 (17)
C3—H3	0.9300	C10—H10	0.9300
C4—C5	1.358 (2)	C11—O3	1.3546 (15)
C4—H4	0.9300	C11—C12	1.3844 (18)
C5—C6	1.4070 (17)	C12—C13	1.3775 (19)
C5—H5	0.9300	C12—H12	0.9300
C6—N2	1.3241 (16)	C13—H13	0.9300
C6—N1	1.3547 (15)	C14—O1	1.2511 (14)
C7—H7A	0.9600	C14—O2	1.2653 (14)
C7—H7B	0.9600	N1—H1	0.8600
C7—H7C	0.9600	N2—H2A	0.8600
C8—C13	1.3873 (17)	N2—H2B	0.8600
C8—C9	1.3887 (16)	O3—H3A	0.97 (2)
			
N1—C2—C3	119.11 (12)	C10—C9—H9	119.2
N1—C2—C7	116.13 (11)	C8—C9—H9	119.2
C3—C2—C7	124.75 (12)	C9—C10—C11	119.92 (11)
C2—C3—C4	119.27 (13)	C9—C10—H10	120.0
C2—C3—H3	120.4	C11—C10—H10	120.0
C4—C3—H3	120.4	O3—C11—C12	117.87 (12)
C5—C4—C3	120.77 (12)	O3—C11—C10	122.92 (11)
C5—C4—H4	119.6	C12—C11—C10	119.21 (12)
C3—C4—H4	119.6	C13—C12—C11	120.25 (12)
C4—C5—C6	119.91 (12)	C13—C12—H12	119.9
C4—C5—H5	120.0	C11—C12—H12	119.9
C6—C5—H5	120.0	C12—C13—C8	121.50 (12)
N2—C6—N1	118.40 (11)	C12—C13—H13	119.2
N2—C6—C5	124.19 (12)	C8—C13—H13	119.2
N1—C6—C5	117.41 (12)	O1—C14—O2	122.68 (11)
C2—C7—H7A	109.5	O1—C14—C8	120.15 (11)
C2—C7—H7B	109.5	O2—C14—C8	117.16 (10)
H7A—C7—H7B	109.5	C6—N1—C2	123.52 (10)
C2—C7—H7C	109.5	C6—N1—H1	118.2
H7A—C7—H7C	109.5	C2—N1—H1	118.2
H7B—C7—H7C	109.5	C6—N2—H2A	120.0
C13—C8—C9	117.58 (11)	C6—N2—H2B	120.0
C13—C8—C14	121.54 (11)	H2A—N2—H2B	120.0
C9—C8—C14	120.88 (11)	C11—O3—H3A	109.1 (12)
C10—C9—C8	121.54 (11)		
			
N1—C2—C3—C4	0.1 (2)	C10—C11—C12—C13	−0.1 (3)
C7—C2—C3—C4	−179.57 (13)	C11—C12—C13—C8	0.3 (3)
C2—C3—C4—C5	0.3 (2)	C9—C8—C13—C12	−0.1 (2)
C3—C4—C5—C6	0.0 (2)	C14—C8—C13—C12	−179.76 (14)
C4—C5—C6—N2	179.79 (13)	C13—C8—C14—O1	6.19 (19)
C4—C5—C6—N1	−0.64 (19)	C9—C8—C14—O1	−173.41 (12)
C13—C8—C9—C10	−0.1 (2)	C13—C8—C14—O2	−174.60 (12)
C14—C8—C9—C10	179.48 (12)	C9—C8—C14—O2	5.80 (18)
C8—C9—C10—C11	0.3 (2)	N2—C6—N1—C2	−179.31 (11)
C9—C10—C11—O3	179.75 (13)	C5—C6—N1—C2	1.10 (17)
C9—C10—C11—C12	−0.1 (2)	C3—C2—N1—C6	−0.82 (18)
O3—C11—C12—C13	179.97 (15)	C7—C2—N1—C6	178.85 (11)

### Hydrogen-bond geometry (Å, º)

**Table:**

*D*—H···*A*	*D*—H	H···*A*	*D*···*A*	*D*—H···*A*
N1—H1···O1^i^	0.86	2.00	2.8499 (13)	169
N2—H2*A*···O2^i^	0.86	1.94	2.7879 (14)	168
N2—H2*B*···O1^ii^	0.86	2.18	2.9902 (14)	157
O3—H3*A*···O2^iii^	0.97 (2)	1.67 (2)	2.6281 (14)	168.5 (19)
C4—H4···O3^iv^	0.93	2.51	3.4134 (17)	163

Symmetry codes: (i) −*x*+1, −*y*+2, −*z*+1; (ii) −*x*+1, *y*−1/2, −*z*+1/2; (iii) −*x*+2, *y*−1/2, −*z*+3/2; (iv) −*x*+2, −*y*+1, −*z*+1.
